# Situational assessment of adult vaccine preventable disease and the potential for immunization advocacy and policy in low- and middle-income countries

**DOI:** 10.1016/j.vaccine.2021.01.066

**Published:** 2021-03-12

**Authors:** Molly Sauer, Prarthana Vasudevan, Ankita Meghani, Karuna Luthra, Cristina Garcia, Maria Deloria Knoll, Lois Privor-Dumm

**Affiliations:** International Vaccine Access Center, Department of International Health, Johns Hopkins Bloomberg School of Public Health, 415 N Washington St, 5^th^ Floor, Baltimore, MD 21231 USA

**Keywords:** Adult immunization, Policy, Implementation, Pneumococcal vaccine, Herpes zoster vaccine, Influenza vaccine

## Abstract

By 2050, the number of adults over 65 years of age will be double the under-5 population, and heavily concentrated in low- and middle-income countries. Population growth and increasing life expectancies call for effective healthy aging strategies inclusive of immunization to reduce the burden of vaccine-preventable diseases, improve quality of life, and mitigate antimicrobial resistance. Based on a review of available literature on the pneumococcal disease, influenza, and herpes zoster epidemiology and economic burden, and the health systems and policy barriers for adult immunization, we identified evidence gaps and considerations for prioritizing adult immunization. The body of evidence for adult immunization and the health and economic burden of adult disease is heavily concentrated in high-income countries. The few countries reporting adult immunization policies generally focus on high-risk groups. Despite robust child immunization programs in most countries, adult immunization programs and policies lag far behind and there is a general lack of appropriate delivery platforms. Global adult disease burden and economic costs are substantial but evidence from low- and middle-income countries is limited. There is a need for a strengthened evidence base and political commitment to drive a comprehensive, global technical consensus on adult immunization.

## 1 Introduction

By 2050, the world’s population of adults aged 65 and older will more than double, from about 700 million in 2019 to over 1.5 billion people [1]. In 2018, the number of adults over 65 years of age surpassed the number of children under 5 years of age for the first time; by 2050, it is estimated that adults over 65 will outnumber children under 5 by two-to-one [1]. The vast majority of these older adults will live in low- and middle-income countries [2]. Global life expectancy has also increased, rising from 61.7 years in 1980 to 72.6 years in 2019 [1], [3]. This trend is expected to continue, with life expectancy rising to 77.1 by 2050, assuming current progress in disease prevention and control continues and catastrophic events do not occur [1].

Thus, there is a need for effective prevention strategies to support healthy aging. Vaccines are one such promising strategy for adults, who may not have developed immunity through vaccination or natural infection and/or who may have waning immunity [4]. As the burden of vaccine-preventable disease (VPD) shifts to older individuals, protecting adults against influenza, pneumococcal disease, herpes zoster, and other VPDs is part of an effective strategy for curbing adult morbidity and mortality, reducing disability, improving quality of life, and protecting against the emergence of antimicrobial resistance—an issue of particular concern for older adults [5], [6].

In 2012, the World Health Assembly (WHA) approved the Global Vaccine Action Plan (2011–2020) to accelerate the benefits of immunization to all children, adolescents, and adults [7], [8]. The WHA endorsed another plan in 2016 that provides a framework within which adult vaccines could be considered and implemented as a priority for health across the life course [9]. Strategies are needed to implement these plans and to encourage global and country-level action. Conceptually, countries are in agreement with the need to implement healthy aging strategies. However, consensus on an adult vaccination agenda eludes the global community.

Advancing political priority hinges on establishing the extent and importance of the issue and the effectiveness of the proposed solution, gaining support from and consensus among key actors, and leveraging key policy windows where advocacy may be most resonant [10]. Previous reviews have aimed to describe the severity of adult VPDs and the cost-effectiveness of vaccination as an intervention. In 2016, Truven Health Analytics, the International Vaccine Access Center (IVAC) at Johns Hopkins University, and the Institute for Health Metrics and Evaluation (IHME) at the University of Washington conducted a narrative review of literature from 2005 to 2015 that reported on pneumococcal disease, influenza, and herpes zoster among adults aged 60 or older and described the epidemiology, economic, health system, and policy barriers for adult immunization [11]. The review noted substantial disease burden for the three target pathogens, but a lack of economic data and awareness of the value of adult vaccination among key stakeholders, including policy makers and health care providers [11]. Reviews conducted in 2013 to evaluate the cost-effectiveness of adult vaccination across priority disease areas have primarily focused on high-income countries and found the evidence is largely generated from ten Western European countries [12], [13]. Economic evaluations in Europe only spanned three VPD areas: influenza, invasive pneumococcal disease (IPD) and pneumococcal pneumonia, and herpes zoster. Despite differences in target cohorts and vaccination strategies, these reviews largely found that the evidence supported the cost-effectiveness of vaccination of adults ≥ 50 years measured against the country-specific standard cost-effectiveness thresholds [12], [14]. A global systematic review of the economic evidence for influenza vaccination of adults found no studies were conducted in low-income countries [15].

Questions remain that impede country decision-making on whether, when, and which vaccines are appropriate for their older adult population. Such decisions are complex; policy makers must weigh not only disease burden and potential health impact, but economic value and impact, quality of life considerations, health systems (i.e., platforms for delivery, incentives to delivery, reimbursement), financing, competing health priorities, and the country’s capacity and political will to consider adult vaccination. Country-level estimates for number of deaths and number of disability-adjusted life years (DALYs) [16] are a vital factor and, ideally, such decisions also consider prevalence of risk factors (i.e. co-morbidities, malnutrition, and environmental pressures), age distribution of the population, public health priorities, and available resources. Disease burden estimation must also consider the indirect effects of vaccinating pediatric populations—reducing disease transmission among vaccinated children can help drive down disease circulation and, thus, reduce disease burden in unvaccinated groups, including adults—as well as the direct effects of adult vaccination.

Additionally, decision-making for potential adult immunization must consider the individual’s functional ability (capability of a person to be and do what they value) and intrinsic capacity (the combined physical and mental capacities on which a person can draw) [17], [18], [19]. These impact vaccine effectiveness by a function of immunosenescence and waning immunity as adults age, and impaired immunity by some of the same factors that affect risk of disease, such as malnutrition and comorbidities [17], [18], [20], [21]. Understanding these factors can help policy makers evaluate the potential need for and impact of an adult vaccination program, particularly one emphasizing functional ability and intrinsic capacity over chronological age [22].

To inform efforts to shape political prioritization of adult immunization and identify barriers and opportunities for its implementation, this rapid situational assessment aims to build upon past reviews and provide a broad overview of some of the key considerations regarding the need for and value of vaccination for older adults. The situational assessment considers available evidence and policies for three priority VPDs—pneumococcus, influenza, and herpes zoster—in order to identify key gaps and cross-cutting issues that may affect implementation of the World Health Organization’s (WHO) Global Vaccine Action Plan and Immunization Agenda 2030, as well as aging and health strategies, particularly in low- and middle-income countries (LMICs).

## 2 Methods

Disease burden modeling conducted by IHME suggested three priority VPDs—influenza lower respiratory tract infections, pneumococcal pneumonia and meningitis, and herpes zoster—were responsible for approximately one in every five communicable disease deaths and DALYs among adults aged ≥ 60 years in 2017 [23]. This rapid situational assessment aims to summarize newly published health and economic evidence and national policy recommendations across these three diseases.

To build upon the Truven review covering 2005–2015, we searched PubMed to identify peer-reviewed English language studies published between January 2015 and August 2017 that reported epidemiological evidence on influenza, pneumococcal disease, or herpes zoster among adults age 50 years and older, expanded from 60 years and older in order to capture potential adult high-risk groups. To extend previous economic reviews, we searched PubMed to identify English language studies published from 2013 through August 2017 that reported economic evidence of the disease burden or vaccination among adults age 50 years and older. Additionally, we included published and unpublished articles, presentations, reports, and abstracts identified from interviews with key experts. Finally, we reviewed publicly available data from the WHO [24] on vaccine introduction and coverage data and published literature on country-level policy and program considerations for adult vaccination.

We provide an initial summary of available evidence on epidemiologic, economic, and policy considerations for vaccination of older adults against these three priority VPDs. This summary aims to identify evidence gaps, help determine if adult immunization policies should be developed or strengthened, and outline barriers to and opportunities for building political prioritization and consensus among scientific and policy experts on the role of immunization in a healthy aging strategy.

## 3 Results

### 3.1 Epidemiology and disease burden of adult VPDs

Of the 370 unique results, 78 met our inclusion criteria. An additional 24 papers were included based on targeted searches and key informant interview recommendations. Among the 102 included papers, 71 were original research articles—six on influenza, 29 on pneumococcal disease (including community-acquired pneumonia [CAP]), 31 on herpes zoster diseases, and five on multiple diseases. Of the 71 original research articles, 63 (89%) described data from studies conducted in one of 25 primarily high- (57 studies) and middle-income (6 studies) countries; six (8%) described data across multiple countries. More than one-third (n = 25, 35%) were conducted in Europe, 17 (24%) in North America, 22 (31%) in Asia-Pacific, and two (3%) in Africa. More studies (n = 12) were conducted in the United States than any other country. The two most commonly studied age groups were adults ≥ 60 years and adults ≥ 65 years.

More than 65% of global deaths, 30% of DALYs, and 32% of years of life lost (YLLs) occur among people 60 years of age or older [23]. Most burden is estimated to be attributed to long-term non-communicable diseases including most cancers, chronic respiratory diseases, and heart disease [25]. However, communicable diseases, including VPDs, contribute to disease burden among older adults [16], [23], [25]. For example, an estimated 3.0 million deaths in adults aged ≥ 60 years were due to lower respiratory, diarrheal, and other common infectious diseases in 2017 [23].

While recommendations for pneumococcal, influenza, and herpes zoster vaccines existed in some countries, coverage levels were generally suboptimal where reported and these three adult diseases remained important contributors to adult disease burden. There is adequate general epidemiological data at the global level to assess the disease burden due to these three VPDs (Table 1) [16], [23], [25], [26]. Globally, total DALY, YLL, and death rates among older adults have been decreasing in the two decades prior to 2017, but high rates persist in parts of sub-Saharan Africa, Asia, and Latin America [11], [16], [23]. Further, the age distribution of these three VPDs may differ in LMIC settings as compared to high-income countries, potentially impacting populations below this assessment’s age cutoff and thus not captured here [27].

**Table 1:** Summary of identified studies describing disease burden and epidemiology of pneumococcus, influenza, and/or herpes zoster among older adults, by World Bank income group.

World Bank Income Group*		Low-income	Lower-middle-income	Upper-middle-income	High-income^1^	Multiple
**Number of studies**		2	2	4	57	6
**Number of countries**		2^2^	2^3^	3^4^	18^5^	–
**Diseases (N studies)**						
	Pneumonia/IPD/CAP^6^	1	2	1	23	2
	Influenza	0	0	1	4	1
	Herpes zoster	1	0	2	26	2
	Multiple	0	0	0	4	1
**High risk groups studied (N studies)**		HIV-infected (1) Hospital (1)	-	Cancer (1)	Autoimmune diseases (2) HIV-infected (2) Chronic illness (1) Aged care facilities (3) Diabetics (2) Cancer patients (2) Hospital (9)	Immunocompromised (1) Hospital (1)

1 Includes Taiwan, per World Bank guideline

2 Malawi (1), Rwanda (1)

3 India (1), Indonesia (1)

4 China (2), Mexico (1), Thailand (1)

5 Australia (4), Belgium (1), Canada (3), Denmark (2), France (3), Germany (1), Italy (4), Japan (5), South Korea (1), New Zealand (2), Norway (2), Poland (2), Spain (5), Sweden (1), Taiwan (5), The Netherlands (3), United Kingdom (1), U.S. (12)

6 IPD = Invasive Pneumococcal Disease; CAP = Community Acquired Pneumonia

To make decisions on vaccination programs, countries often seek disease burden data at the country level. Although regional [28] or sub-national estimates for some of these diseases are available for some countries, aggregate national estimates are limited. Only the U.S. estimated national incidence of influenza, pneumococcal disease, and herpes zoster disease among adults ≥ 50 years [29]. There were significant data gaps in LMICs, with most studies conducted in high-income settings like the U.S. [30], Canada [31], Australia [32], and Japan [33]. This may be more pronounced for some VPDs than others; for example, herpes zoster studies were almost exclusively focused in high-income settings including Poland [34], the U.S. [35], [36], and France [37], [38]. Of the studies we found in LMICs, most describe the epidemiology of the cases (e.g., pneumococcal serotype distribution), not the disease burden, and were conducted in large cities and therefore may not be representative of the country or region more broadly. In some settings, data from national registry inputs, which are frequently used by high-income countries (HICs) to obtain adult disease data, may be unreliable or nonexistent [39].

IHME’s Global Burden of Disease study is the only study we found that provides comprehensive estimates of deaths and disability attributable to these three adult VPDs and others; few other independent published studies provide global estimates for specific VPDs among adults over age 50 or other age ranges beyond 50 years (e.g., 60+, 65 + ) [25], [26], [40].

In order to prevent disease, vaccines need to be administered before the onset of disease, thus age is an important consideration from a policy and implementation perspective. Any recommendation will be reliant on the body of evidence to support an age target (and possibly cut-off) for vaccination, which may differ between HICs and LMICs. Age-specific data on immunosenescence, risk factors, and vaccine performance are critical to inform vaccination strategies in adults. Many studies identified only evaluated adults at ≥ 60 years; evidence to consider younger age targets (e.g. ≥ 50 years) is lacking.

### 3.2 Economic evidence for adult immunization

The search yielded 319 studies, of which 52 met inclusion criteria (Table 2). Among these, only three WHO regions were represented—Americas (N = 21, 40%), Europe (N = 21, 40%), and the Western Pacific (N = 10, 19%)—with the U.S. contributing the most evidence from any one country (N = 16, 31%). The majority of studies were on influenza (N = 24, 46%) and IPD and/or pneumococcal pneumonia (N = 20, 38%), with three studies spanning more than one VPD (influenza and pneumococcal) [41], [42], [43]. The majority of evidence was cost-effectiveness analyses (CEA) (N = 33, 63%), with additional evidence from cost of illness studies (N = 9, 17%) and cost analyses (N = 6, 12). The most common comparator in CEAs was a different existing vaccine (N = 15, 45%), with 13-valent pneumococcal conjugate vaccine (PCV13) vs. 23-valent pneumococcal polysaccharide vaccine (PPSV23) (N = 8, 40%) and quadrivalent inactivated influenza vaccine (IIV4) or high-dose trivalent inactivated influenza vaccine (IIV3-HD) vs. trivalent inactivated influenza vaccine (IIV3) (N = 7, 29%) being the most common comparators. The age groups included in these studies were generally consistent with all studies including adults aged ≥ 65 years, and 35% including aged ≥ 50 years. However, reporting of age-specific outcomes when age-specific inputs were used was inconsistent, and 11 studies were excluded from the final analysis because the older adult population could not be extracted from the aggregated outcomes.

**Table 2:** Summary of identified studies describing economic burden and/or vaccine impact for pneumococcus, influenza, and/or herpes zoster among older adults, by WHO region.

WHO Region		Africa (AFRO)	Americas (AMRO/PAHO)	Eastern Mediterranean (EMRO)	Europe (EURO)	South-East Asia (SEARO)	Western Pacific (WPRO)	Total
**Number of studies**		0	21	0	21	0	10	52
**Number of countries**^1^		0	5	0	16	0	4	25
**Age groups represented**^2^								
	≥50 years	–	6	–	11	–	2	18
	≥60 years	–	3	–	3	–	2	7
	≥65 years	–	15	–	12	–	7	34
**Vaccines assessed**^3^		–	PPSV23 PCV13 IIV3 IIV3-HD IIV3-adjuvanted IIV4 HZV	–	PPSV23 PCV13 IIV3 IIV4 HZV	–	PPSV23 PCV13 IIV3 IIV4	PPSV23 PCV13 IIV3 IIV3-HD IIV4 HZV
**Diseases (N studies)**								
	Pneumonia/ IPD	–	9^5^	–	9	–	2	20
	Influenza	–	12^5^	–	5	–	7	24
	Herpes zoster	–	3	–	7	–	1	11
**Types of studies (N studies)**								
	Cost-effectiveness analysis (CEA)	–	13^6^	–	15^6^	–	5	33^6^
	Cost of illness (COI)	–	3	–	3	–	3	9
	Cost-utility analysis (CUA)	–	1	–	–	–	–	1
	Cost analysis (CA)	–	3	–	1	–	2	6
	Budget impact analysis (BIA)	–	2^6^	–	3^6^	–	–	5^6^
**Comparator category for CEA (N studies)**^4^								
	Delivery and uptake strategies	–	5	–	2	–	1	8
	No vaccination	–	4	–	9	–	1	14
	Other vaccine	–	7	–	5	–	3	15
	Target cohorts	–	1	–	4	–	1	6

1 Americas: Brazil, Canada, Colombia, Panama, United States; Europe: Belgium, Czech Republic, France, Finland, Germany, Hungary, Israel, Italy, Kazakhstan, Netherlands, Poland, Romania, Spain, Sweden, Ukraine, United Kingdom; Western Pacific: Australia, China (incl. Hong Kong), Japan, South Korea

2 Age groups counted separately if study included multiple age groups results (e.g., 50–64, ≥65 years)

3 PCV13 = Pneumococcal conjugate vaccine (13-valent); PPSV23 = Pneumococcal polysaccharide vaccine (23-valent); IIV3 = Trivalent influenza vaccine (and includes vaccines given in standard dose and high-dose); IIV4 = Quadrivalent influenza vaccine; HZV = Herpes-zoster vaccine

4 Studies can include multiple comparator categories (e.g., different vaccines and no vaccination); Target cohorts compare different age groups or ‘at risk’ groups

5 Three studies included both pneumococcal and influenza

6 Two studies (1 AMRO/PAHO, 1 EURO) included both a cost-effectiveness analysis and budget impact analysis

Studies generally concluded that vaccination with pneumococcal conjugate vaccines (PCV), influenza vaccines, or herpes zoster vaccines was largely cost-effective or cost-saving, with incremental cost effectiveness ratios ranging from dominant—meaning the intervention is “clinically superior and cost-saving” compared to the alternative [44]—to £257,771 per quality-adjusted life-year (QALY) [45], [46].

For all three VPDs assessed, the economic evaluation methods used varied significantly across settings, limiting generalizability. Although each vaccine has its own unique challenges in evaluating its economic burden and impact, a few limitations are consistent challenges for all vaccines. Only ten studies collected data from patient interviews or hospital records, while all others used secondary data from published and unpublished literature, national surveillance networks, or health insurance claims databases. Because medical costs are sensitive to the case definition used, cost estimates must be carefully matched to the disease burden parameters used; the disease burden and cost parameters often came from different sources, so this was often not possible. Although hospitalized and severe cases were consistently included across studies, distinctions between private and public facilities and the case definitions for outpatients and non-care-seeking cases, such as the definition of influenza-like illness or community-acquired pneumonia were not always specified [47], [48]. Country and regional differences in the cost of care and productivity loss, particularly among HICs and LMICs, makes generalizing results across settings problematic.

Most studies used a third-party payer or health system perspective focusing on outpatient and hospitalized visits, and despite approximately half of the studies adopting a societal perspective, few included non-medical or out-of-pocket expenditures. Indirect costs were included in 41% of studies, but the methodology for estimating productivity loss varied across settings. Others studies chose not to account for productivity loss because the targeted older adult population was of retirement age [49] or because of data paucity [50]. Information on the vaccine program costs were not disaggregated into common categories (e.g., cold chain, health worker personnel), limiting comparison between different health systems. Outside of the five budget-impact studies [51], [52], [53], [54], [55], few studies discussed the financial implications of new vaccine introduction.

These methodological differences, which can result in an over or underestimate of the true costs and lead to incorrect conclusions about vaccine cost-effectiveness, were largely a result of a lack of available data. For influenza, the primary data limitation is due to seasonal and annual variation. Studies that assessed cost-effectiveness of influenza vaccine over multiple seasons found the vaccine to be cost-effective in some years but not others, largely due to variability in disease severity and vaccine efficacy [56], [57]. For pneumococcal and herpes zoster vaccines, vaccine effectiveness and waning immunity among older adults was unknown or based on assumptions. Herpes zoster vaccine study methods varied between studies that assumed some level of waning immunity occurs compared to those that did not, but generally adult vaccination among ages ≥ 50 years or ≥ 60 years were considered to be cost-effective.

Inclusion of indirect effects in vaccine economic models remains the area with greatest reliance on assumptions. Few studies assessing influenza used dynamic models accounting for changing transmission patterns because of data availability limitations [48]. Only one study considered the impact of PCV13 vaccination in adults ≥ 65 years on disease transmission among adults > 50 years of age [55], and most studies that considered herd immunity considered only the impact of childhood vaccination with PCV13 in high-income country settings [46], [58], [59], [60], [61]. Seven studies of PCV chose not to account for indirect effects, assuming no net change in disease incidence among adults because of serotype replacement, an issue seen mainly with PCV7 versus PCV10 or PCV13 [42], [47], [53], [54], [55], [62], [63]. While most studies found PCV13 vaccination in the elderly to be cost-effective, the incremental cost effectiveness ratios were sensitive to both vaccine effectiveness, herd immunity estimates, and coverage.

For all three focal VPDs, adult vaccination was generally considered cost-effective and cost-saving compared to no vaccination, but few studies were conducted in LMICs and none in Africa or South-East Asia. Despite previous recommendations [64], most studies fail to incorporate the broader impact of vaccination in adults, including averting catastrophic health expenditures to households, labor gains, life expectancy gains, and benefits of human capital investment.

### 3.3 Global and regional policies and recommendations

**Fig. 1:**
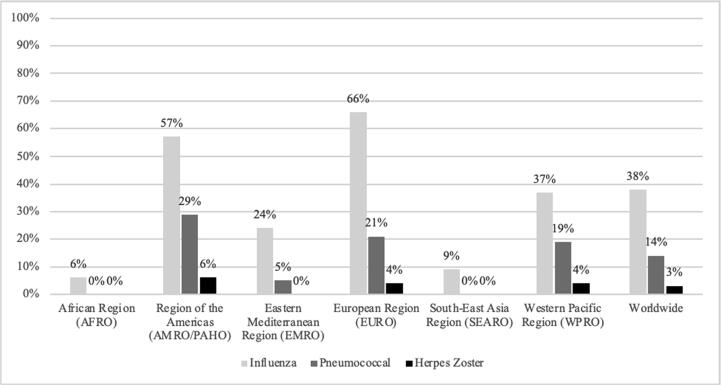
Percent of WHO Member States with national vaccination policies for influenza, pneumococcal disease and varicella for individuals 15 years of age and older, 2017 (24).

Based on the available 2017 data in the WHO Monitoring System Database [24], 38% of the 194 WHO Member States reported a national adult vaccination policy for influenza, 27% for pneumococcal disease, and 3% for zoster (Fig. 1).

Among these countries, influenza vaccination policies generally target adults ≥ 60 or ≥ 65 years of age, with fewer targeting those ≥ 50 years or ≥ 55 years. Policies focused on high-risk groups, such as the elderly or adults with chronic diseases, pregnant women, healthcare providers, travelers (including those on the annual Hajj pilgrimage), and airport staff. Most countries with a national influenza vaccination policy were HICs from the Americas or Europe [65]. In contrast, only 6% and 9% of countries from Africa and South-East Asia, respectively, reported a national influenza vaccination policy.

Similarly, for pneumococcal vaccination, a higher percentage of countries in the Americas (29%) and Europe (21%) reported national policies for adults. Compared to influenza, fewer countries reported a national policy for pneumococcal vaccination. These policies generally targeted adults ≥ 60 years or ≥ 65 years. Although a few countries targeted “risk groups,” they were not clearly defined or lacked standardization among definitions; some considered risk groups as adults ≥ 50 or 65 years, medically at-risk, belonging to indigenous populations, or young men. Almost all policies recommended PPSV23; only one country recommended PCV exclusively. As of mid-2017, no countries in Africa or South-East Asia reported the presence of a national adult vaccination policy for pneumococcal disease [24].

For herpes zoster vaccination, only five countries—Canada, the U.S., France, Israel, and Australia—reported a national policy in 2017 [24]. The policies targeted adults ≥ 60 years, with Australia targeting adults ≥ 80 years. In Canada, the policy was only implemented in Ontario; similarly, in France, the policy was sub-national. Though not reported in the WHO monitoring system, the United Kingdom has also implemented a herpes zoster vaccination program; in place since 2013, it targets adults ≥ 70 years, with additional age groups specified for a catch-up campaign [66]. As of 2017, no countries from the Africa, Eastern Mediterranean, or South-East Asia regions reported a herpes zoster vaccination policy [24].

Despite the presence of robust child immunization programs in most countries, adult immunization programs and policies have received much less attention, particularly in LMICs. Countries recommending and scaling up adult immunization programs tend to be wealthier and have stronger governance structure and capacity for vaccine decision-making, as well as robust immunization systems and vaccine delivery platforms [65]. As indicated by a recent global review of national immunization policies, these countries also have more experience introducing new or under-utilized vaccines [65].

Studies suggest that global institutions are influential in countries’ vaccination policy decision-making [67], [68], [69]. For many countries, WHO recommendations may drive the decision to introduce national immunization policies. In 2012, the WHO published guidelines on seasonal influenza vaccination, with pregnant women as a priority group; other high-risk groups were also identified, including young children, individuals with chronic diseases, the elderly, and healthcare workers [70]. As of 2017, the WHO had not yet recommended introducing herpes zoster or pneumococcal vaccines for adults. Without published guidelines on recommendations for adult immunization, nor a clear consensus on a preferred adult immunization strategy, countries may be less likely to address the issue independently.

Factors influencing national vaccine policy decision-making may differ by a country’s wealth status. Although cost-effectiveness studies have been key in all settings, data priorities likely differ between LMICs and HICs [65]. In LMICs, greater need and emphasis is often placed on capturing VPD burden among adults, assessing the mortality impact of interventions, and calculating their affordability and return on investment [71], [72]. In India, for example, data on VPD burden, vaccine efficacy and safety based on in-country studies, and operational costs will be key to inform decision making [71], [72]. In HICs, data on economic productivity and averting complications in high-risk groups have been more pertinent for policy discussions [65]. A survey of HICs found that, despite the cost-effectiveness of vaccines, recommendations were generally made for certain sub-groups of the adult population [73]. These different approaches highlight the need to understand factors that influence policy decisions and mechanisms by which decisions and processes may be better informed.

The lack of comprehensive epidemiological data on VPD burden among adults has delayed adult vaccination policy development, particularly in LMICs [71], [72], [74]. Without evidence of unmet need, many countries have been challenged to build political commitment for adult immunization [10]. Robust surveillance allows for better understanding of shifting disease epidemiology and informs future vaccination policies, especially for vaccines selected based on regional serotype prevalence or for diseases which have not been previously measured. Surveillance data may also be used to identify high-risk or priority groups and benchmark progress against national adult immunization coverage targets [74]. For example, Saudi Arabia’s extensive surveillance system has been key for identifying and minimizing the health risks associated with the annual Hajj pilgrimage, resulting in seasonal influenza and pneumococcal vaccination recommendations for pilgrims, as well as required proof of meningococcal vaccination prior to obtaining an entry visa [75], [76], [77], [78], [79], [80].

In other countries, pregnant women, healthcare providers and older adults have been identified as high-risk groups. The U.S. Advisory Committee on Immunization Practices (ACIP) recommends all health care providers be vaccinated against ten pathogens, including seasonal influenza [81], [82]. Several countries in the Americas, Europe, and the Western Pacific have introduced similar policies requiring influenza vaccination for healthcare providers [24], [65].

Determining the patient profile and time period in which to vaccinate, ideally prior to a decline in intrinsic and functional capacity, is also complex and will require consideration of factors beyond chronological age [22]. To efficiently use health resources, countries need vaccines that better address immunosenescence and strategies (e.g., schedule adjustments) that target the right populations at the right time [83]. To this end, a Working Group on Metrics and Research Standards on Healthy Ageing was established in March 2017 to develop a plan to measure intrinsic and functional capacity [84].

## 4 Discussion

Our rapid situational assessment identified several gaps in the existing body of evidence for adult immunization, and opportunities to improve policies and programs to reduce the burden of adult VPDs (Box 1).

**Box 1:** Gaps and recommendations to advance adult immunization policies and advocacy.

**GAPS**
- Epidemiologic and economic literature is primarily from HICs - Few LMICs have implemented policies or recommendations for influenza, pneumococcal or herpes zoster vaccination - Little is known about disease burden for adults under 60 years of age - Disease burden and economic data are generally viewed through a narrow lens of deaths and direct costs of disease - Country policies are self-reported and data are unreliable - Surveillance capacity for adult VPD is lacking in LMIC - There is no comprehensive WHO recommendation for adult immunization - Vaccine delivery systems are not optimized for adult immunization

**RECOMMENDATIONS**
**Strengthen the body of evidence through research** - Assess country-level adult VPD burden data in LMICs - Assess VPD burden data for adults < 60 years - Standardize age strata and definitions for epidemiologic and economic analyses of adult VPDs - Establish the impact of community protection from pediatric immunization programs in varying settings and identify key population gaps not addressed by these programs - Assess the impact of adult VPD on quality of life, functional capacity, and productivity, and the potential gains from immunization - Establish epidemiological and cost parameters from LMICs to better inform economic analyses, including understanding out-of-pocket expenditures on adult vaccines - Assess key drivers of country decision-making for vaccination policy, to inform further studies **Improve data quality and availability** - Refine global reporting mechanisms (e.g., Joint Reporting Form) to improve the accuracy and completeness of data on country-level vaccination policies and recommendations - Monitor immunization coverage using quality measures **Establish policy and advocacy guidance** - Advance global and regional recommendations for adult immunization, including key considerations for countries - Promote immunization standards through professional bodies - Develop communication messages that resonate with older adults and their caregivers about the value of older adult immunization with existing and future new vaccines. **Assess and address health system and delivery gaps** - Assess the feasibility of expanding immunization financing mechanisms to include adult vaccination and mitigate out-of-pocket expenses - Conduct research to understand the benefits and challenges of integrating adult immunization and primary care - Expand access to vaccinations to non-medical places, such as pharmacies, workplaces, and schools/colleges; particularly in the context of public health emergencies (e.g., COVID-19), more innovative delivery should be explored (e.g., self-administration of mobile outreach)

While the burden of influenza, pneumococcal disease, and herpes zoster among older adults is substantial, regional and country pictures are not as rich and evidence of burden in LMICs is lacking. Resources will need to be directed towards ensuring a greater awareness of the adult burden of disease and the potential impact of adult immunization programs.

Further vaccine impact modeling is needed to assess the value of adult vaccination and impact on quality of life—cornerstones of a healthy aging strategy. Future research should also consider health system implications, including integrating immunization into primary health care and the role of both primary and non-primary healthcare providers, such as pharmacists. Given that factors such as the proportion of adults with co-morbidities and risk factors can influence incidence, details on the country-specific assumptions used to produce disease burden estimates are needed to assess the level of confidence in the estimates at a country level. Consideration of how available studies handle uncertainty in estimation will be important to further evaluate and synthesize the body of evidence and define the research, policy, and advocacy agenda for vaccination of older adults.

As age-specific incidence in LMICs is not necessarily the same as for HICs, future work must clarify the assumptions made about age distribution of the disease. The lack of epidemiologic data for older adults below 60 years of age presents a challenge in identifying what, if any, differences exist by age to better inform policymakers on the appropriate age for a vaccination policy. For pneumococcal disease, herd effects of child vaccination play a key role in reducing adult disease burden; quantifying any additional benefits of vaccinating adults directly in this context is an important area needing further study to help inform the evidence base and national and global policies.

Current economic evidence relies heavily on epidemiological and cost parameters from HICs, which may not be applicable to LMIC settings [48], [85], [86], [87], [88]. Within the available evidence, differences in model parameters and assumptions make generalizing the cost-effectiveness of different vaccination strategies using different vaccine types or targeting different populations difficult. Economic assessments will benefit from evidence from LMICs on vaccine efficacy among adults ≥ 50 years, changing serotype or strain circulation, and transmission patterns resulting from adult and childhood vaccination. In countries systems are not currently established to deliver adult immunization, the structural and cost issues of delivering vaccines are important considerations, particularly as this may serve as a platform for other services or could be integrated into existing platfoms. While studies from HICs benefited from existing surveillance systems as a source of data, similar surveillance systems are lacking in many LMIC studies, and none of the studies included the cost of enhanced surveillance programs in the cost-effectiveness calculation. Further research is needed in these areas to more accurately quantify the economic costs and benefits of adult vaccination, as well as understand reimbursement and incentives for providers to offer vaccines.

To monitor adult immunization policies, thorough and timely reporting by countries is critical yet often suboptimal. While the data reported to the WHO monitoring system may be incomplete, the adult vaccination policy disparities by country wealth category is striking. Expanding surveillance and implementation capacity to enable sustainable influenza vaccination programs and policies—particularly in LMICs, where a significant gap remains—is a critical need, and is supported by a range of key partners. As the vaccine product landscape expands and new, potentially more affordable or easier to administer vaccines become available, more countries may consider and potentially implement adult vaccination policies [89], [90], [91].

Improving the availability and quality of evidence on the health and economic burden and impact of adult VPDs—particularly in LMICs, which will soon comprise the vast majority of over-60 adults by mid-century—is critical to developing scientific, policy, and advocacy strategies that can effectively address this important public health issue.

## Declaration of Competing Interest

The authors declare the following financial interests/personal relationships which may be considered as potential competing interests: [Lois Privor-Dumm has previously received funding from drug companies for investigator driven policy research (Pfizer, GSK and Merck). This does not alter our adherence to *Vaccine* policies on sharing data and materials. All other authors declare that they have no known competing financial interests or personal relationships that could have appeared to influence the work reported in this paper.].
